# Opioid receptor distribution in the claustrum-dorsal endopiriform complex

**DOI:** 10.1016/j.isci.2026.116213

**Published:** 2026-06-02

**Authors:** Matthew Bolger, Jesse Jackson, Anna M.W. Taylor

**Affiliations:** 1 Department of Pharmacology, University of Alberta, Edmonton, Alberta, Canada; 2 Neuroscience and Mental Health Institute, University of Alberta, Edmonton, Alberta, Canada; 3 Cancer Research Institute of Northern Alberta, University of Alberta, Edmonton, Alberta, Canada; 4 Department of Physiology, University of Alberta, Edmonton, Alberta, Canada; 5 Department of Anesthesiology and Pain Medicine, University of Alberta, Edmonton, Alberta, Canada

**Keywords:** biological sciences

## Abstract

The claustrum and dorsal endopiriform form a subcortical structure reciprocally connected to the neocortex and enriched in opioid receptors. However, the precise cellular distribution of opioid receptors within this region remains unclear. Using multiplexed fluorescent *in situ* hybridization we mapped the expression of mu (*Oprm*), delta (*Oprd*), kappa (*Oprk*), and ORL1 (*Oprl*) opioid receptors in the mouse claustrum-dorsal endopiriform. *Oprk* expression was restricted to excitatory neurons and enriched in a single population of *Synpr*+ claustrum core projection cells. In contrast, *Oprd*, *Oprm*, and *Oprl* genes were more broadly distributed across excitatory and inhibitory populations. Spatial mapping further confirmed *Oprk* expression was restricted within the claustrum core. Analysis of a publicly available snRNA-seq dataset of the macaque claustrum also revealed a similar receptor distribution. This demonstrates an evolutionarily conserved, cell-type-specific organization of opioid receptor expression in the claustrum-dorsal endopiriform and suggests nuanced roles for other opioid receptors in modulating claustrocortical circuits.

## Highlights

• Each opioid receptor exhibits a unique distribution in the claustrum-dorsal endopiriform
• This cell-type-specific opioid receptor organization is conserved across mice and macaques
• KOR (Oprk) expression uniquely defines excitatory claustrum core projection neurons

## Graphical abstract

**Figure:**
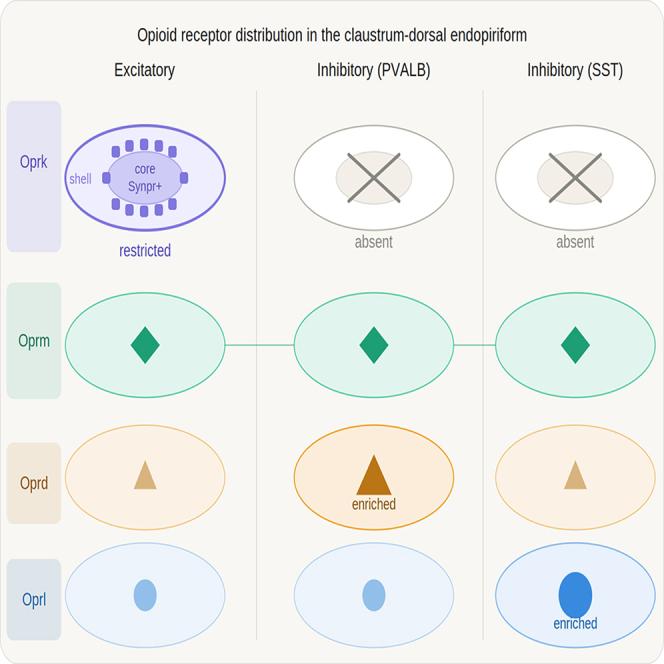

## Introduction

The claustrum and dorsal endopiriform regions comprise a thin sheet of subcortical gray matter that sits between the putamen and insula. This region spans the rostro-caudal axis of the brain and is reciprocally connected to much of the neocortex, particularly the frontal and associative cortices.^1^^,^^2^ It also receives bottom-up inputs from subcortical limbic structures, which positions the claustrum-dorsal endopiriform complex to be a central hub in cortical network integration and cognition. Activation of this pathway coordinates cortical activity during online and offline states, and has been linked to behaviors associated with opioid signaling including pain,^3^^,^^4^^,^^5^ opioid reinforcement/relapse,^6^^,^^7^ and stress.^8^ This region is enriched in opioid receptor expression; however, the exact cellular distribution of mu (MOR), delta (DOR), kappa (KOR), and opioid receptor like-1 (ORL-1) opioid receptors is unknown. A fulsome understanding of the distribution of opioid receptors within the claustrum-dorsal endopiriform complex will reveal ways in which opioid signaling influences cortical network dynamics in health and disease.

The claustrum and endopiriform are made up of excitatory projection cells and several populations of inhibitory cells defined by peptide content, including parvalbumin, neuropeptide Y and somatostatin. Excitatory projection cells are topographically organized based on cortical projection targets^9^^,^^10^ and neurochemical profiles.^11^^,^^12^^,^^13^ While the identification of the rodent claustrum-dorsal endopiriform complex has been historically quite challenging due to the thin structure and lack of obvious anatomical borders from adjacent cortical regions, recent gene sequencing data have identified a number of genes (*Synpr*, *Nr4a2*, *Lxn*) that have been validated markers of these regions.^12^^,^^14^^,^^15^^,^^16^^,^^17^ Early studies examining opioid receptor binding and gene/protein expression have reported particularly high expression of ORL-1 (*Oprl*) and KOR (*Oprk*) in the claustrum-dorsal endopiriform region, with more modest expression of DOR (*Oprd*) and MOR (*Oprm*).^18^^,^^19^^,^^20^^,^^21^^,^^22^^,^^23^ More recent single cell sequencing experiments in mice have described *Oprk* and *Oprm* expression in excitatory claustrum cells, with relatively lower expression of *Oprd*.^24^ Electrophysiological recordings of opioid agonist-evoked activity in the claustrum found MOR, DOR, and KOR agonists reduced activity in claustrum projection cells; however, only KOR agonists reduced monosynaptic transmission.^24^ This difference in activity suggests a wider, more nuanced distribution of opioid receptors beyond excitatory cells. We do not yet know the precise cellular and spatial distribution of all four opioid gene transcripts across all neuronal populations within the claustrum-dorsal endopiriform region.

Using multiplexed fluorescence *in situ* hybridization (mFISH) we sought to determine the distribution of the four opioid receptors at a single cell resolution in the claustrum-dorsal endopiriform. We then applied hierarchical cluster analysis to describe the expression of opioid receptors within excitatory and inhibitory clusters in the claustrum and dorsal endopiriform. Using a publicly available dataset of claustrum gene expression in mice and macaques, we determined the opioid expression pattern was similar between mice and non-human primates. Overall, these data indicate a distinctive and evolutionarily conserved pattern of expression of opioid receptors across the claustrum-dorsal endopiriform complex.

## Results

### Classifying claustrum cells visualized by mFISH

**Figure 1:**
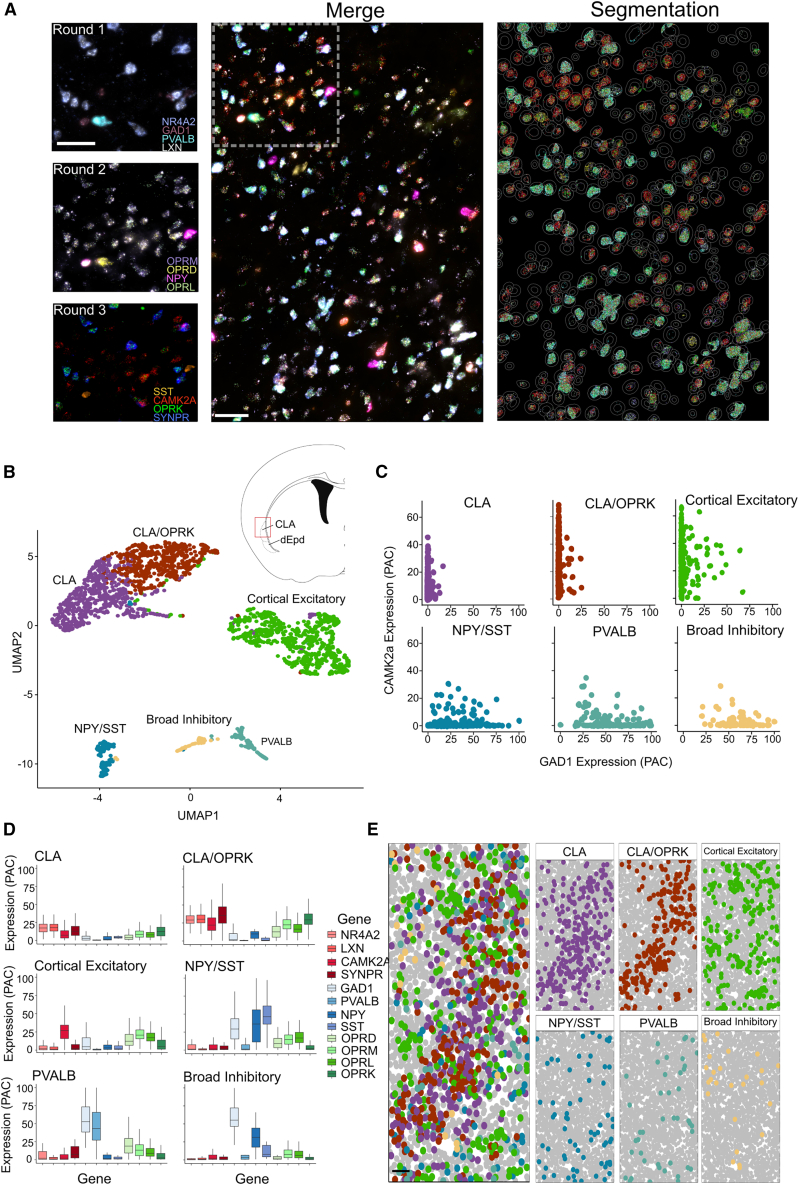
Identification of claustrum neuronal populations utilizing multiplexed fluorescent *in situ* hybridization (A) Representative images of the claustrum. Labeling for all 12 probes occurred in three iterative steps on the same brain slice. After each step, images were acquired (left) and spatially registered images were merged into one 12-channel image (middle). A cell segmentation tool was used to define regions of interest that were used for analysis (right). Scale bars, 50 μm. (B) UMAP based non-linear dimensionality reduction of processed claustrum cell expression data, labeled and colored as per hierarchical clustering assignment. (C) *Camk2a* expression plotted against *Gad1* expression for each identified cluster. PAC = percent area covered and indicates the fraction of the cell occupied by fluorescently tagged gene probe. (D) Expression levels of all genes for each identified claustrum cell cluster. (E) Cells identified in (B) plotted using scaled spatial coordinates. Scale bars, 50 μm.

mFISH was used to describe opioid receptor distribution in the claustrum-dorsal endopiriform complex at a single cell resolution. Several classical markers of excitatory and inhibitory cells were chosen to orient opioid receptor expression to specific subclasses of neurons. Calcium/calmodulin dependent protein kinase 2a (*Camk2a*) was used to identify broad excitatory cells, with nuclear receptor related protein 1 (Nurr1; gene *Nr4a2*), latexin (*Lxn*), and synaptoporin (*Synpr*) used as specific markers of claustrum-dorsal endorpiriform excitatory cells. Glutamate decarboxylase 1 (*Gad1*) was used to identify inhibitory cells, and parvalbumin (*Pvalb*), neuropeptide Y (*Npy*), and somatostatin (*Sst*) used to demarcate specific inhibitory populations. The expression of these 12 genes was spatially registered across sections of the claustrum and dorsal endopiriform (Figure 1A). The percent area covered (PAC) by each fluorescently tagged probe in each cell was calculated to compare gene expression levels across cells.

Through hierarchical clustering analysis, 6 patterns of marker co-expression were identified in the claustrum (Figure 1B). Three of the identified clusters were enriched with the excitatory gene *Camk2a* (claustrum excitatory cluster [CLA], CLA/OPRK, cortical excitatory), while three were enriched with the inhibitory gene *Gad1* (NPY/SST, PVALB, broad inhibitory). To further confirm this broad classification, the level of *Gad1* expression within each cluster was compared against the expression of *Camk2a* (Figure 1C). Clusters with high expression (over 50%) of *Camk2a* (CLA, CLA/OPRK, and cortical excitatory) had less than 1.5% coverage by *Gad1* (OPRK/CLA = 0.38%, cortical excitatory = 1.36%, CLA = 0.21%). Conversely, clusters with high expression (over 75%) of *Gad1* had less than 4% coverage by *Camk2a* (NPY/SST = 3.05%, Pvalb = 3.74%, broad inhibitory = 3.21%). This indicates that *Camk2a* and *Gad1* expression reliably identifies segregated populations of putative excitatory and inhibitory cells. Of the cells identified in the claustrum, approximately 85% belonged to excitatory clusters and 15% belonged to inhibitory clusters.

Two of the excitatory clusters prominently expressed all three claustrum markers (*Synpr*, *Nr4a2*, and *Lxn*) indicating two distinct subpopulations of claustrum excitatory cells. However, one of these subpopulations exhibited higher marker expression across the board, with the two most differentially expressed markers being *Synpr* and *Oprk*. This population was classified as a CLA/OPRK cell cluster while the former was classified as the CLA cell cluster. These two excitatory claustrum clusters also exhibited notable spatial distributions, with the CLA/OPRK cells clustering toward the core of the claustrum and the CLA cells more widely distributed across the core and shell of the claustrum (Figure 1E). The third excitatory cluster did not significantly express any claustrum markers but expressed spatial and transcriptomic properties of cortical excitatory cells. As such it was classified as “cortical excitatory” and likely represents generalized excitatory cells expressed across the neocortex.

Of the cells classified as inhibitory (*Gad1*+), the largest cluster was enriched in *Sst* and *Npy*. The other major inhibitory cluster prominently expressed *Pvalb*. The final, smaller, population prominently expressed only *Gad1*, with *Npy* being expressed to a lesser extent. There were no identifiable spatial distribution patterns within the inhibitory cells likely due to a lack of cells compared to the excitatory clusters.

### Opioid receptor expression varies by claustrum cell type

**Figure 2:**
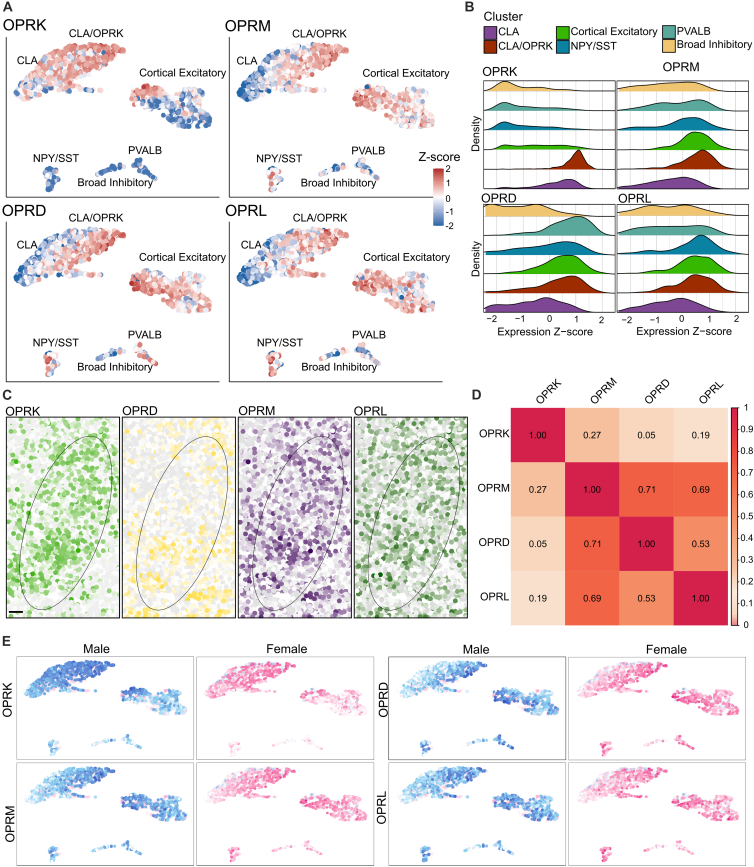
Opioid receptor expression within the claustrum (A) Labeled UMAP projection colored as per each cell’s expression of each opioid receptor individually. (B) Stacked density plots of opioid receptor expression within each identified cluster. (C) Spatial plot as in Figure 1E but colored as per each cell’s *Z* score of opioid receptor expression. Claustrum location approximated based on the position of putative claustrum cells (CLAs and CLA/OPRK clusters) and indicated with an ellipse indicating the region covered by 80% of the CLA-specific excitatory cells. Scale bars, range from −2 to +2, Colored from gray to assigned color. (D) Pearson correlation table for the opioid receptor expression across all claustrum cells. (E) UMAP of opioid receptor expression from male and female brains. Cells colored as per sex of animal, blue (male) or pink (female). Scale bars, range from −2 to +2, colored from white to assigned color, cells of opposing sex present but with reduced opacity.

We next assessed the expression of all four opioid receptors within the identified clusters (Figures 2A and 2B). KOR (*Oprk*) exhibited a distribution within the observed cell populations distinct from the other opioid receptors. *Oprk*, was most prominently expressed within the excitatory CLA/OPRK cluster. *Oprk* expression was detectable in other excitatory clusters (CLA and broad excitatory), but at significantly lower levels than the CLA/OPRK cluster. Notably, *Oprk* expression was not detectable above background in any of the inhibitory clusters. In contrast, expression of the other opioid receptor genes exhibited much wider distribution across both inhibitory and excitatory clusters. *Oprd* expression was highest in the PVALB inhibitory cluster; however, it was also present consistently within the cortical excitatory, CLA/OPRK and NPY/SST cell populations. *Oprl* expression was highest in the NPY/SST cluster; however, it was also present consistently within the cortical excitatory and CLA/OPRK clusters. *Oprm* expression was also found in both excitatory and inhibitory clusters but expressed at higher levels within most excitatory populations. Apart from *Oprk* and the CLA/OPRK cluster, no other opioid receptor gene was sufficient to define a specific cluster or cell type.

Of all the opioid receptor genes, *Oprk* was most restricted to the region identified as the claustrum (defined by *Nr4a2* expression, Figure 2C). Cells expressing *Oprd*, *Oprm*, *or Oprl* showed a wider spatial distribution that extended beyond the claustrum into the neighboring cortex. *Oprk* expression was the least correlated with other opioid receptor genes (Figure 2D; Figure S1), indicating that cells expressing *Oprk* are less likely to also express other opioid receptor genes. In contrast *Oprm*, *Oprl*, *and Oprd* expression were more consistently correlated with each other, with *Oprm* expression showing the highest degree of correlation with the other opioid receptors. This indicates that cells that express *Oprm* are more likely to express at least one other opioid receptor gene (besides *Oprk*).

Finally, opioid receptor distribution in the claustrum was compared between male and female tissue (Figure 2E; Figure S2). No significant differences in opioid receptor expression were noted in the majority of cell populations. The only exception was *Oprd* expression within the CLA/OPRK population, which was higher in females than males. However, given the limited sample size we interpret these results with a degree of caution. While these results suggest little difference between sexes, more work is required in the future to make a conclusive determination.

### Dorsal endopiriform cell populations and opioid receptor distribution is similar to those of the claustrum

**Figure 3:**
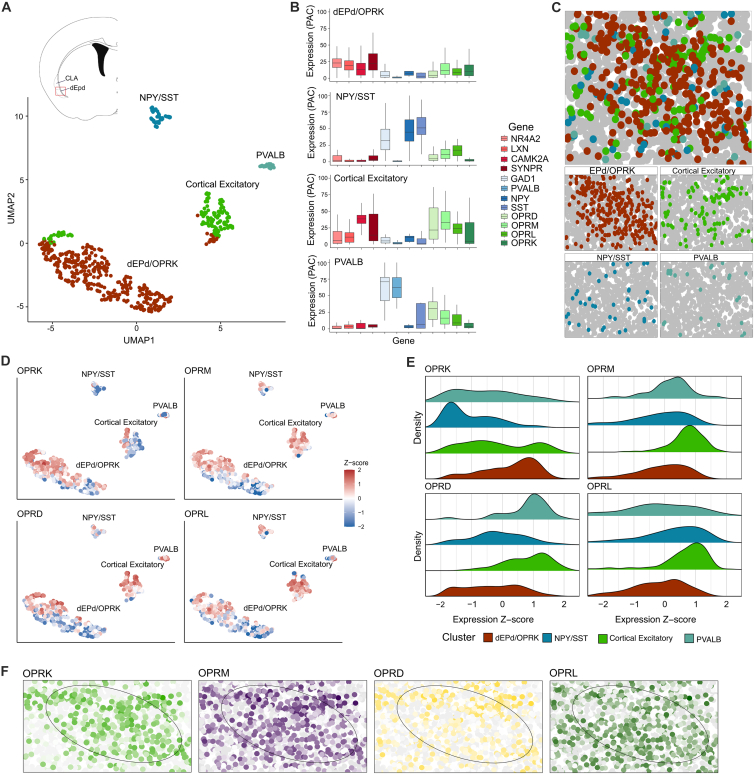
Opioid receptor expression in the dorsal endopiriform (A) UMAP based non-linear dimensionality reduction of processed endopiriform cell expression data, labeled and colored as per assigned cluster. (B) Expression levels of all genes for each identified endopiriform cell cluster. (C) Cells identified from (A) plotted using scaled spatial coordinates. (D) Labeled UMAP projection colored as per each cell’s expression of each opioid receptor individually. (E) Stacked density plots of opioid receptor expression within each identified cluster. (F) Spatial plot as in (C) but colored as per each cell’s *Z* score of opioid receptor expression. Endopiriform location approximated based on position of excitatory dorsal endopiriform cells (dEPd/OPRK cluster) and indicated with an ellipse indicating 80% of the region covered by dorsal endopiriform excitatory cells. Scale bars, range from −2 to +2, colored from gray to assigned color.

The dorsal endopiriform is adjacent to the claustrum and shares common anatomical and neurochemical features. This region has been defined as the extended claustrum and can be identified based on expression of Nurr1 (*Nr4a2*).^14^ Here, we compared the cellular distribution of opioid receptors within the dorsal endopiriform and compared this to the distribution in the claustrum (Figure 3). Using the same data analysis pipeline as before, we found 585 cells with significant neuronal marker expression. These cells formed four clusters which were similar to those populations defined in the claustrum (Figures 3A and 3B). The first identified cluster was an excitatory cluster which showed high expression of *Nr4a2*, *Lxn*, *Synpr*, and *Oprk*. This population was defined as the dEPd/OPRK cluster and was analogous to the CLA/OPRK population defined in the claustrum. While *Oprk* expression within this dorsal endopiriform population was comparatively lower than the claustrum, these cells still represented the primary *Oprk*+ cell population. Like their claustrum analogs, this cluster was spatially distributed toward the core of the dorsal endopiriform (Figure 3C).

The next identified cluster was also deemed excitatory due to the prominent expression of *Camk2a* (cortical excitatory). The cells within this cluster expressed relatively less *Nr4a2* and *Lxn* and therefore lack defined markers for the claustrum-dorsal endopiriform region. This suggests this population represents a broad cortical excitatory population analogous to the cortical excitatory population defined in the claustrum. This identity is further supported by the pattern of opioid receptor expression, in which it is enriched in *Oprm*, *Oprd*, and *Oprl* genes with relatively lower levels of *Oprk* expression (Figures 3D and 3E).

The remaining two clusters were classified as inhibitory based on their high levels of *Gad1*. The NPY/SST cluster was notable for its high expression of *Npy* and *Sst* (Figures 3A and 3B). Much like in the claustrum, this cluster was enriched with *Oprl*, while *Oprk* was largely unexpressed (Figures 3D and 3E). The PVALB cluster was defined by the high level of *Pvalb* expression, and consistent with the claustrum, was enriched with *Oprd*. No distinguishable spatial distribution was observed (Figure 3C). There were no notable sex differences in opioid receptor expression within any dorsal endopiriform cell population (Figure S3).

Overall, the opioid receptor distribution in the dorsal endopiriform was consistent with the patterns observed in the claustrum. *Oprk* expression was largely limited to excitatory cells that express claustrum-dorsal endopiriform markers such as *Nr4a2*, *Lxn*, and *Synpr*. Meanwhile the other opioid receptor subtypes showed no such specificity for excitatory cells. Like in the claustrum, high expressing *Oprk* cells tended to be concentrated together in the core region of the dorsal endopiriform. No other opioid receptor subtype showed any identifiable spatial trends.

### Macaque claustrum

**Figure 4:**
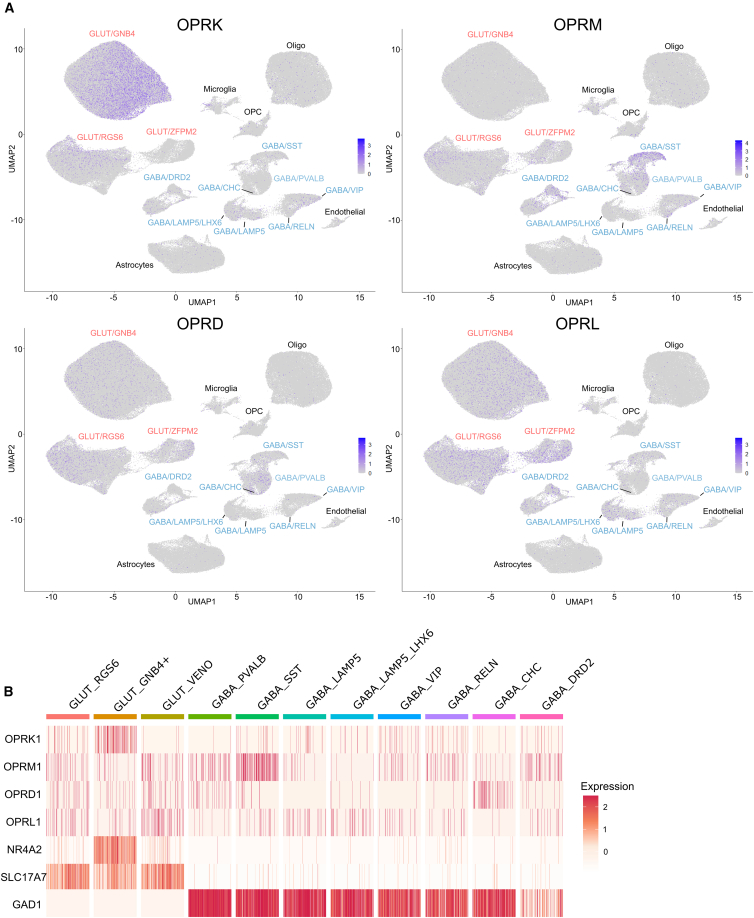
Opioid receptor expression in the macaque claustrum (A) UMAP based representation of opioid receptor expression within all identified macaque claustrum neuron populations. Cells colored based on level of expression represented through pearson residuals. Excitatory clusters labeled in red, inhibitory clusters labeled in blue. (B) Gene expression heatmap of both opioid receptors and select population markers within identified neuronal populations down sampled to 1,000 cells.

The claustrum is a highly conserved structure with analogous regions in the human and non-human primate.^11^^,^^31^ Using publicly available single nucleus sequencing datasets of the mouse and macaque claustrum,^11^ we described the distribution of all four opioid receptors within defined clusters of neuronal cells. In both mouse and macaque datasets, *Oprk* expression was enriched in a subpopulation of excitatory cells defined by *Gnb4* expression (Figure 4). In the macaque claustrum, this represented the largest excitatory cell class, whereas in the mouse claustrum, Gnb4+ cells comprised a smaller excitatory population, accounting for approximately 15% of glutamatergic neurons (Figure S4). In the macaque claustrum, *Oprk* expressing cells accounted for approximately a third of the *Gnb4* cluster, while in the mouse, more than 90% of excitatory cells expressed *Oprk* (Figure S4). In contrast, *Oprk* expression was low across inhibitory cell clusters in both species.

Similar to the mFISH analysis of the mouse, the sn-RNA seq dataset of the mouse and macaque showed the expression of the other opioid receptors was less population specific. In both datasets, *Oprm* expression was prominently seen within inhibitory populations, especially the *Sst* subpopulations. Within macaque excitatory cells, *Oprm* was most prominent within the population of deep layer 6 insular cells (GLUT/ZFPM2). *Oprd* and *Oprl* expression exhibited a similar bias toward this population, although they were more widely expressed across all excitatory populations. This contrasted with the mouse expression of *Oprm*, *Oprd*, and *Oprl*, which showed little bias to any single excitatory population. Within inhibitory cell populations, *Oprd* was preferentially expressed in both PVALB and chandelier (CHC) neurons in both mouse and macaque. In the macaque, *Oprl* was expressed across most of the cell populations at consistent levels, with both classes of parvalbumin inhibitory neurons being the only place where *Oprl* was largely absent. This was largely consistent with the mouse claustrum as well, with elevated expression *Oprl* within somatostatin neurons being the only notable difference. Results from our mFISH analysis aligned with that of the sn-RNA seq dataset and provide a more comprehensive understanding of opioid receptor distribution and support the premise that this distribution is largely conserved across species.

## Discussion

The claustrum-dorsal endopiriform complex is a region enriched in opioid receptors. However, the exact cellular and spatial distribution of all four opioid receptors was unknown. Here, we used multiplexed fluorescent *in situ* hybridization and spatial transcriptomics to describe the pattern of expression of MOR, DOR, KOR, and ORL1 receptors in the claustrum-dorsal endopiriform complex.

In line with previous gene and protein expression studies, all four opioid receptors were detected with the claustrum and dorsal endopiriform. *Oprk* expression was distinctive in that it was the only receptor that was restricted to excitatory neurons. *Oprk* expression was highest in clusters containing defined markers for claustrum cells, and lower in the neighboring cortical clusters. This is in line with other studies that indicates KOR expression is highest in the claustrum relative to neighboring cortical regions.^5^^,^^11^ Previous studies have identified two spatially distinct projection cell populations within the claustrum termed “core” and “shell,” with core cells projecting to frontal/midline cortical structures and shell cells projecting preferentially to posterior structures.^7^^,^^8^^,^^9^^,^^10^ Core claustrum projection cells can be identified based on their high expression of *Nr4a2*, *Lxn*, and *Synpr*.^12^ Here, we found *Oprk* was most highly expressed within the CLA cluster enriched with *Synpr* (CLA/OPRK). This cluster had relatively higher levels of *Nr4a2* and *Lxn* than the other CLA, suggesting *Oprk* expression is most highly expressed on claustrum core projection cells. This was confirmed with spatial analysis that found CLA/OPRK cells clustered toward to the core of the claustrum. The expression of *Oprk* in core claustrum projection cells is supported by several other lines of evidence. For example, previous sequencing experiments have reported that *Oprk* is enriched in a single excitatory population.^13^^,^^24^ Nurr-1 (*Nr4a2*) is a key regulator guiding the development of the claustrum core cell molecular phenotype.^14^ When Nurr-1 was knocked down in the claustrum, *Oprk* expression was significantly downregulated.^14^ While a nuanced classification of dorsal endopiriform projection cells based on neurochemical or anatomical projection targets has not yet been described, our spatial transcriptomics assessment reveals similar distribution of *Oprk* to the claustrum, where it was enriched in the excitatory endopiriform cluster (dEpd/OPRK). Overall, these results indicate KOR receptors are expressed on a subpopulation of core claustrum-dorsal endopiriform excitatory cells. While the functional ramifications of this distribution remain to be studied, the preferential expression on core cells suggests KOR ligands will more strongly influence claustrum output modules targeting frontal/midline cortical regions.

*Oprd*, *Oprl*, and *Oprm* exhibited a much wider distribution across the excitatory and inhibitory clusters within the claustrum and dorsal endopiriform. This has been shown to have functional implication for how opioid agonists impact claustrum function. For example, while KOR agonists abolished optogenetically evoked excitatory activity in the claustrum, MOR and DOR agonists only reduced slower, long latency recurrent excitatory responses.^24^ While *Oprd* and *Oprl* expression was similarly expressed within all excitatory populations, these genes showed dissociable patterns of distribution within the inhibitory subpopulations. While *Oprd* expression was highest in the *Pvalb* inhibitory cluster (PVALB), *Oprl* expression was highest within the *Sst* inhibitory cluster (NPY/SST). Similar to the spatial distribution of claustrum excitatory neurons described above, inhibitory populations are also arranged in a mosaic core/shell topography with *Sst* inhibitory cells located within the claustrum shell and *Pvalb* inhibitory cells within the claustrum core.^10^ This indicates that *Oprd*, located preferentially on *Pvalb+* cells, will have a stronger disinhibitory impact on core claustrum cells that project to frontal midline regions over neurons in the claustrum shell projecting to posterior cortical regions. In contrast, *Oprl* expression was highest within the *Sst* population that indicates it will preferentially impact shell projection cells.

We also analyzed publicly available single nucleus sequencing datasets of both the mouse and macaque claustrum.^11^ In both mouse and macaque datasets, *Oprk* expression was enriched within a subpopulation of claustrum projection cells (*Gnb4+*). Primates exhibited a unique polarization of *Oprk* expression within excitatory cells, with an expansion of Oprk-GNB4 cells compared to the mouse (which were almost entirely Oprk+) (Figure S4). These Oprk-/Gnb4 cells form a unique output module whose projection targets overlap very little with those of the Oprk+/GNB4 cells.^11^ The functional implications of this cytoarchitectural organization remain to be determined. Like in the mouse, the other opioid receptors exhibited a wider distribution across most inhibitory and excitatory populations, with no obvious cellular or spatial distribution.

Overall, these data show a conserved, cell-type-specific organization of opioid receptors across the claustrum-dorsal endopiriform complex. All four opioid receptors were highly expressed within the region, and most cell populations expressed more than one opioid receptor. Notably, only the KOR gene, *Oprk*, emerged as a defining molecular feature of excitatory claustrum core projection neurons, indicating KOR signaling will selectively influence a subset of claustrum output modules targeting frontal cortical regions.

### Limitations of the study

As previously mentioned, the claustrum lacks well-defined anatomical boundaries. This makes distinguishing the claustrum core, claustrum shell and surrounding insula a non-trivial process. While multiple molecular markers exist to this end, the limited number of probes allowed by mFISH meant we could not use markers specific for either claustrum shell cells or cortical cells. Furthermore, there exists a number of inhibitory populations within the claustrum we could not specifically identify, which limits our insight into opioid receptor expression within these populations to the publicly available sn-RNA seq datasets. Finally, the relatively smaller number of dorsal endopiriform cells compared to claustrum cells led to the formation of less well-defined clusters and a less robust analysis overall. While there were clear similarities to the claustrum, further work could be done to elucidate the extent to which opioid receptor distribution within the dorsal endopiriform relates to that of the claustrum.

## Resource availability

### Lead contact

Further inquiries or requests for materials, procedures, or datasets should be directed to and will be fulfilled by the lead contact, Anna Taylor (ataylor1@ualberta.ca).

### Materials availability

This study did not generate new unique reagents.

### Data and code availability

• mFISH image analysis datasets generated in this study can be found at https://doi.org/10.5281/zenodo.17633672.
• This study does not report original code.
• Any additional information required to reanalyze the data reported in this study is available from lead contact upon request.

## Acknowledgments

The authors would like to thank Mark Cembrowski for helpful discussions and technical guidance, as well as Christian Faig, Julia Nickols, and members of the Jackson lab for comments on the manuscript. A.M.W.T. is supported by the 10.13039/501100000001Alberta Cancer Foundation Chair in Palliative Care and a Canada Research Chair Tier 2 in Pain and Addiction. This work was supported by the 10.13039/501100000038Natural Sciences and Engineering Research Council of Canada (RGPIN 2018-04010 to A.M.W.T.) and the 10.13039/501100000024Canadian Institutes of Health Research (PJT 192040 to A.M.W.T.; PJT 195864 to J.J.). Microscopy work was supported the University of Alberta Faculty of Medicine & Dentistry Cell Imaging Core, which receives financial support from the Faculty of Medicine & Dentistry, the 10.13039/501100018911University Hospital Foundation, Striving for Pandemic Preparedness – The Alberta Research Consortium, and Canada Foundation for Innovation (CFI) awards to contributing investigators. Imaging assistance provided by Kiera Smith and Dr. Hilmar Strickfaden.

## Author contributions

Conceptualization, A.M.W.T. and J.J.; methodology, A.M.W.T., J.J., and M.B.; investigation, M.B; writing – original draft, M.B.; writing – review and editing, A.M.W.T., J.J., and M.B.; visualization, M.B.; supervision and funding acquisition, A.M.W.T. and J.J.

## Declaration of interests

The authors declare no competing interests.

## STAR★Methods

### Key resources table

**Table:** 

REAGENT or RESOURCE	SOURCE	IDENTIFIER
**Critical commercial assays**		

RNAscope Hiplex v2 Assay (488, 550, 650, 750)	ACDBio	https://acdbio.com/rnascope-hiplex-assays
Mouse: C57BL/6	The Jackson Laboratory	JAX# 000664

**Deposited data**		

Raw CLA/dEPd mFISH Data	This Paper	https://doi.org/10.5281/zenodo.17633672
Mouse and Macaque Claustrum snRNA-seq data	Lei et al.^11^	https://www.braindatacenter.cn/datacenter/web/#/dataSet/details?id=1904344961189928962

**Software and algorithms**		

LAS X	Leica Microsystems	https://www.leica-microsystems.com/products/microscope-software/p/leica-las-x-id/
Qupath	Bankhead et al.^27^	https://qupath.github.io/
Stardist	Schmidt et al.^28^	https://github.com/stardist/stardist
ImageJ	Schindelin et al.^25^	https://imagej.net/software/fiji/
R (4.5.0)	The R Foundation	https://www.r-project.org/
HyperStack Reg	Ved Sharma^26^	https://github.com/ved-sharma/HyperStackReg
Seurat	Hao et al.^32^	https://satijalab.org/seurat/
Caret	Max Kuhn^30^	https://cran.r-project.org/web/packages/caret/index.html

### Experimental model and study participant details

#### Animals

Male and female C57B16/J mice, acquired from Jackson Laboratories (Bar Harbor ME) were used for this study. Four mice (23 weeks) were used in total, 2 male and 2 female. Mice were housed in ventilated plastic cages with standard bedding, on a 12-h light/dark cycle, with free access to standard rodent chow and water. All experimental procedures were performed in compliance with the Canadian Council on Animal Care’s Guidelines and Policies with approval from the University of Alberta Health Sciences Animal Care and Use Committee (AUP #2493).

### Method details

#### Tissue preparation

Mice were euthanized using sodium pentobarbitol (Euthasol). Following a transcardiac perfusion of ice-cold saline, brains were taken and immediately immersed in cryo-embedding medium (OCT). After being cooled over dry ice, brains were moved to a −80°C freezer for storage. Brains were sectioned at a thickness of 12 μm using a cryostat tissue slicer and mounted on glass slides. 1–2 slices per animal were collected for analysis.

#### Multiplexed fluorescent *in situ* hybridization data acquisition

mFish assay was performed as per RNAscope HiPlex12 v2 assay user manual (Advanced Cell Diagnostics) starting with the fresh frozen tissue pretreatment. Probes for mFish were purchased from Advanced Cell Diagnostics and include: *Nr4a2* (423351-T1), *Pvalb* (421931- T2), *GAD1* (400951- T3), *Lxn* (585801- T4), *Oprd* (427371- T5), *Oprm* (315841- T6), *Npy* (313321- T7), *Oprl* (514301- T8), *Sst* (404631 - T9), *Camk2a* (445231- T10), *Oprk* (316111- T11), *Synpr* (500961- T12). All 12 probes with unique tails (T1-T12) were hybridized to the tissue and following 3 successive amplification steps the first set of cleavable fluorophores were hybridized (T1-T4). Tissue was then counterstained with DAPI and coverslipped with ProLong Gold antifade mounting medium. Following image acquisition slides were decoverslipped by soaking in a saline sodium citrate buffer and fluorophores (T1-T4) were cleaved. This process was repeated for 2 more rounds using fluorophores (T5-T8) and (T9-T12). Each probe within a round was conjugated to a distinct fluorophore: AF488(T1,T5,T9), Dylight550(T2,T6,T10), Dylight650(T3,T7,T11), AF750(T4,T8,T12). After each round of hybridization, z stack images (0.5 μm step size) were captured using a Leica THUNDER-Deconvolution Widefield Microscope using a 63x oil immersion objective.

During the first imaging session the claustrum-endopiriform complex region was identified through the expression of both *Nr4a2* and *Lxn*. For ease of analysis, the claustrum and dorsal endopiriform were captured separately. 4–9 contiguous images of the claustrum or dorsal endopiriform regions were captured and stitched together to form a single composite image. Maximum intensity projections from each round were loaded into Fiji^25^ and merged using the HyperStackReg plugin.^26^Transformation matrices were generated through rigid body transformation applied to DAPI images from rounds 2 and 3 to align them with that of round 1. Merged images were then loaded into Qupath^27^ to be pseudo colored and analyzed. Cell segmentation was performed on the DAPI channel of each merged image with the StarDist2D Qupath plugin using the pretrained model “dsb2018_heavy_augment.pb”.^28^ The following parameters were used: probability threshold: 0.67, cell expansion: 5, cell constrain scale: 2, and pixel size: 0.5. This defined each cell’s nucleus and approximated the cytosol as a region of interest within each image. Probe expression was then detected within each region of interest utilizing the Qupath Subcellular detection function. Appropriate intensity thresholds were determined by identifying the mean channel intensity within a given image then adding a standard number of standard deviations depending on the marker. Additional parameters for inhibitory markers and *Synpr*: minimum spot size: 0.5, max spot size: 2, expected size: 0.7. Remaining markers: minimum spot size: 0.3, max spot size: 1, expected: 0.5. The pixel area of detected fluorescent puncta within each region of interest were converted to um^2^, grouped by channel and summed. Cell/probe detection results from each merged image were combined and exported to R (version 4.5.0) for computational analysis.

#### Multiplexed fluorescent *in situ* hybridization analysis

6,155 nuclei were registered across all mFish images of the claustrum-dorsal endopiriform complex. Probe expression was normalized using a probe’s percent area coverage (PAC) of its region of interest (the cell).^29^ Linear dimensionality reduction (PCA) was performed and principal components representing approximately 95% of variance were extracted. From these principal components, the Euclidean distance between cells was calculated and the resultant distance matrix was used for hierarchical clustering (ward.d2). Cells with DAPI expression but no clear expression of any of the 12 probes were excluded from analysis. Lack of probe expression precludes our ability to identify these cells and likely represent non-neuronal cells or unlabeled neurons. The remaining 2,269 cells were re-clustered and used for subsequent cluster-based analysis. To visualize these results, nonlinear dimensionality reduction (UMAP) was performed on the previously extracted principal components using the umap package (Manhattan method, all other parameters default). Cells represented through the umap projection were colored as per their assigned cluster identity. The resulting visualization showed cluster assignments which were consistent with their relative position in the UMAP projection.

To assess the spatial distribution of different cell types, spatial plots were generated from the data of 5–6 images merged into a single plot. Images from both male and female animals were included in each merged plot. Each object (cell) generated through cell segmentation had their pixel coordinates along with marker expression levels and cluster affiliation recorded. Cells previously eliminated from cluster analysis were included as part of a separate cluster for spatial accuracy and visualizations of marker expression. As the images of the claustrum varied in size and orientation, location coordinates were transformed to be between 0 and 1 using the caret R package.^30^ This allowed cells from different images to be plotted on the same axis while retaining their original spatial conformation. In a subset of plots, an ellipse generated from cells belonging to claustrum (CLA and CLA/OPRK) or dorsal endopiriform (dEpd/OPRK) related clusters were drawn at a confidence level of 0.8.

### Quantification and statistical analysis

Boxplots were generated to visualize the distribution of gene expression in cells of a given cluster. Boxes represent the interquartile range with the center line being the median PAC for a given marker. Whiskers show distribution to at most 1.5× the interquartile range and outlier points are not shown. To more effectively contrast the level of gene expression between clusters, the *Z* score of each markers’ log(x+1) normalized PAC within a given cell was calculated. These standardized values were used to color points on a gradient scale according to their level of gene expression in both UMAP and spatial plots. The Pearson coefficient was calculated for each pair of transcripts across all detected neurons to determine the degree of colocalization between opioid receptors and across inhibitory and excitatory clusters. An unpaired Wilcox test was used to investigate any potential sex differences in opioid receptor expression within a given cluster. Statistical significance was set at *p* < 0.05.

Processed sn-RNA seq datasets of the mouse and macaque claustrum were accessed online through: https://www.braindatacenter.cn/datacenter/web/#/dataSet/details?id=1904344961189928962. The seurat package in R was used to manipulate and visualize gene expression data. Cluster assignments and dimensionality reduction remained unchanged from initial analysis.^11^^,^^32^
