# Supplementary material for: Opioid receptor distribution in the claustrum-dorsal endopiriform complex
Source: iScience. 2026 Jun 2;29(6):116213. doi: 10.1016/j.isci.2026.116213 (PMC13254841; doi:10.1016/j.isci.2026.116213)
Supplement: Document S1. Figures S1–S4 [file mmc1.pdf]

iScience, Volume *29*

Supplemental information

Opioid receptor distribution in the claustrum-dorsal endopiriform complex

Matthew Bolger, Jesse Jackson, and Anna M.W. Taylor

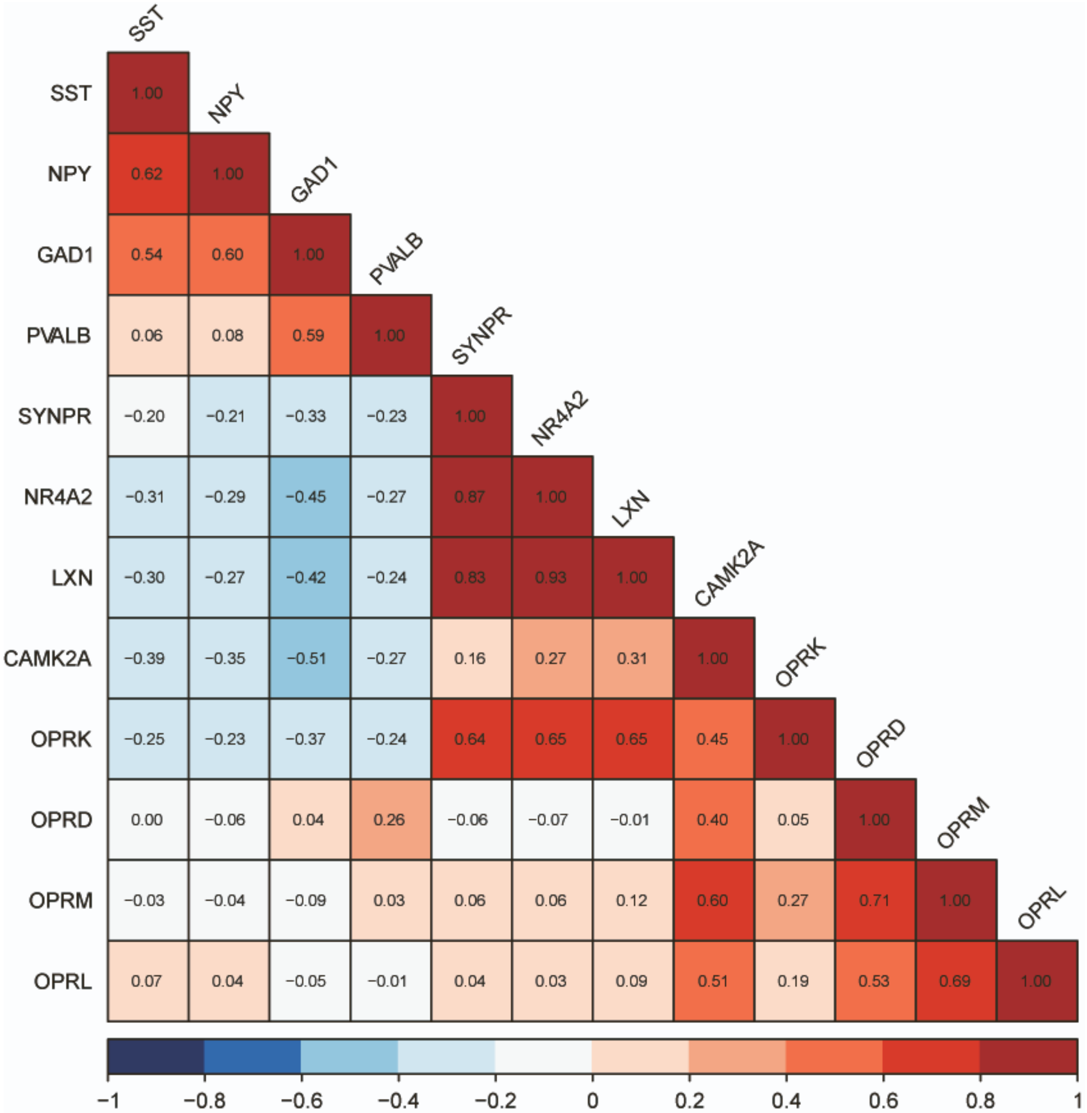
SST
NPY
GAD1
PVALB
SYNPR
NR4A2
LXN
CAMK2A
OPRK
OPRD
OPRM
OPRL
SST 1.00
NPY 0.62 1.00
GAD1 0.54 0.60 1.00
PVALB 0.06 0.08 0.59 1.00
SYNPR −0.20 −0.21 −0.33 −0.23 1.00
NR4A2 −0.31 −0.29 −0.45 −0.27 0.87 1.00
LXN −0.30 −0.27 −0.42 −0.24 0.83 0.93 1.00
CAMK2A −0.39 −0.35 −0.51 −0.27 0.16 0.27 0.31 1.00
OPRK −0.25 −0.23 −0.37 −0.24 0.64 0.65 0.65 0.45 1.00
OPRD 0.00 −0.06 0.04 0.26 −0.06 −0.07 −0.01 0.40 0.05 1.00
OPRM −0.03 −0.04 −0.09 0.03 0.06 0.06 0.12 0.60 0.27 0.71 1.00
OPRL 0.07 0.04 −0.05 −0.01 0.04 0.03 0.09 0.51 0.19 0.53 0.69 1.00
−1 −0.8 −0.6 −0.4 −0.2 0 0.2 0.4 0.6 0.8 1

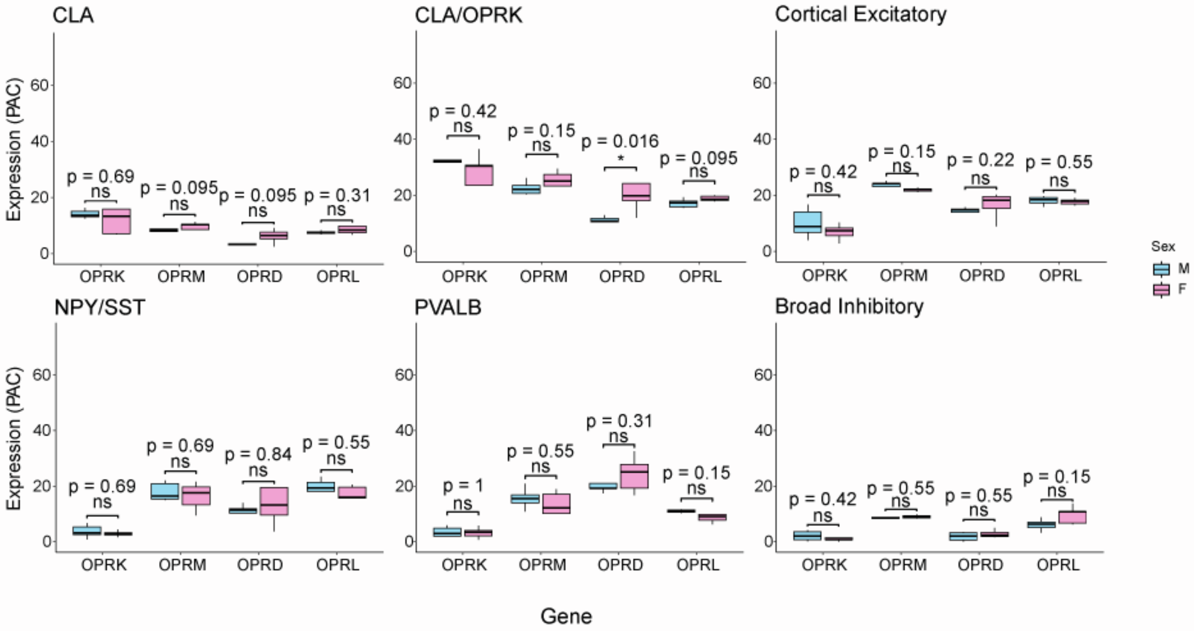
CLA
CLA/OPRK
Cortical Excitatory
NPY/SST
PVALB
Broad Inhibitory
Expression (PAC)
OPRK
OPRM
OPRD
OPRL
p = 0.69
p = 0.095
p = 0.095
p = 0.31
p = 0.42
p = 0.15
p = 0.016
p = 0.095
p = 0.42
p = 0.15
p = 0.22
p = 0.55
p = 0.69
p = 0.69
p = 0.84
p = 0.55
p = 1
p = 0.55
p = 0.31
p = 0.15
p = 0.42
p = 0.55
p = 0.55
p = 0.15
ns
*
Sex
M
F
Gene

A

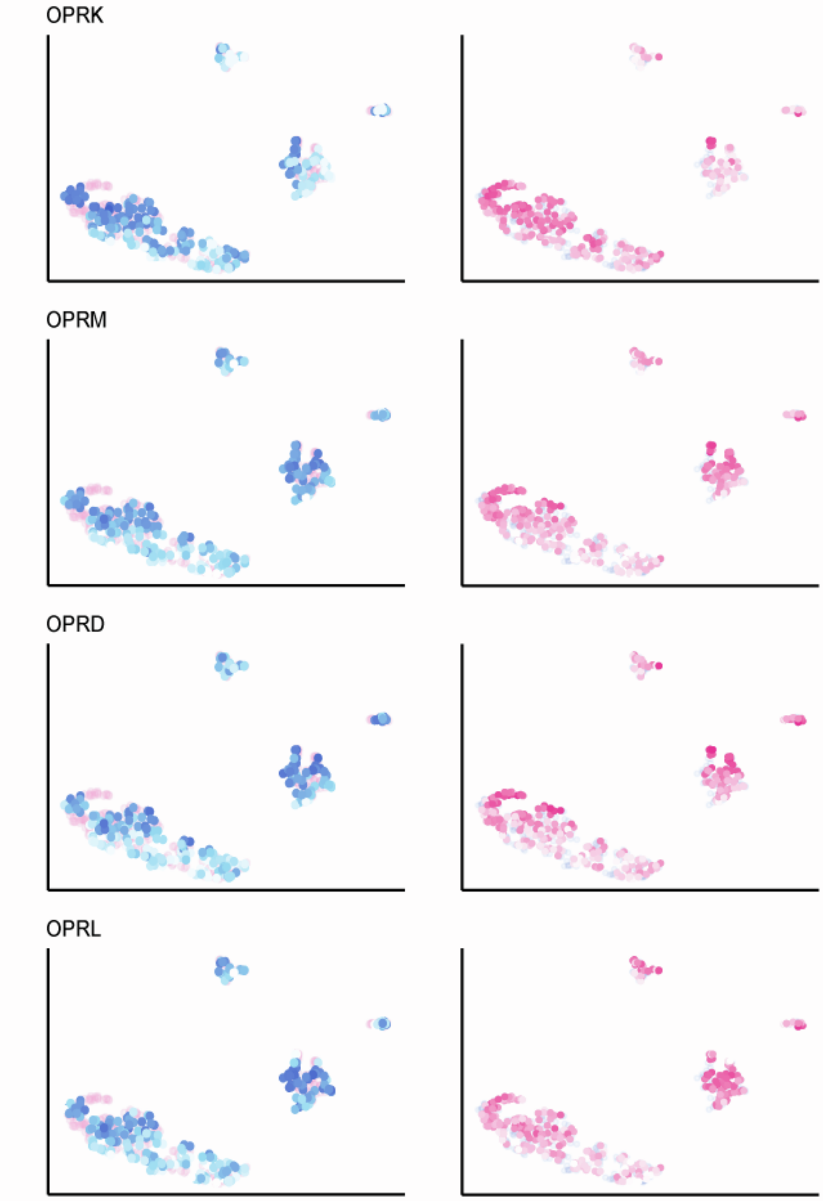

OPRK
OPRM
OPRD
OPRL

B

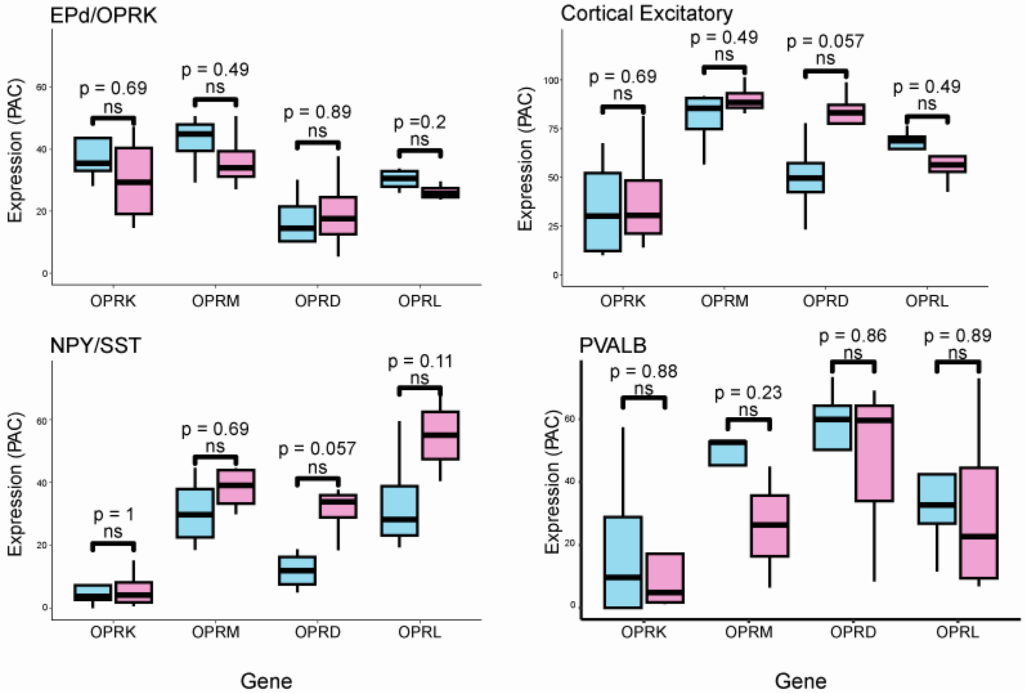

EPd/OPRK
p = 0.69
ns
p = 0.49
ns
p = 0.89
ns
p =0.2
ns
Expression (PAC)
OPRK
OPRM
OPRD
OPRL
Cortical Excitatory
p = 0.69
ns
p = 0.49
ns
p = 0.057
ns
p = 0.49
ns
Expression (PAC)
OPRK
OPRM
OPRD
OPRL
NPY/SST
p = 1
ns
p = 0.69
ns
p = 0.057
ns
p = 0.11
ns
Expression (PAC)
OPRK
OPRM
OPRD
OPRL
Gene
PVALB
p = 0.88
ns
p = 0.23
ns
p = 0.86
ns
p = 0.89
ns
Expression (PAC)
OPRK
OPRM
OPRD
OPRL
Gene

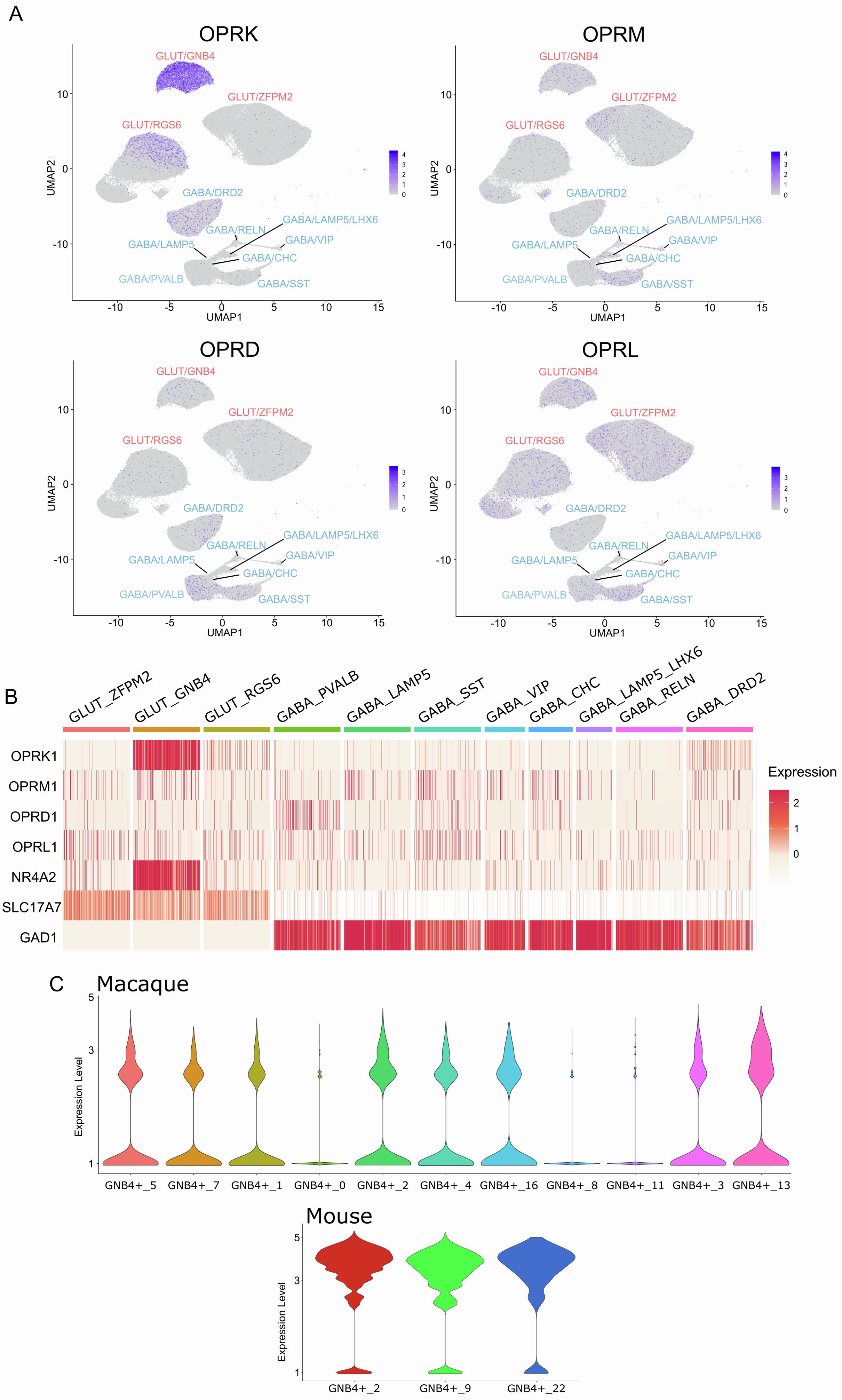
A
OPRK
OPRM
OPRD
OPRL
GLUT/GNB4
GLUT/ZFPM2
GLUT/RGS6
GABA/DRD2
GABA/LAMP5/LHX6
GABA/RELN
GABA/VIP
GABA/LAMP5
GABA/CHC
GABA/PVALB
GABA/SST
UMAP1
UMAP2
B
GLUT_ZFPM2
GLUT_GNB4
GLUT_RGS6
GABA_PVALB
GABA_LAMP5
GABA_SST
GABA_VIP
GABA_CHC
GABA_LAMP5_LHX6
GABA_RELN
GABA_DRD2
OPRK1
OPRM1
OPRD1
OPRL1
NR4A2
SLC17A7
GAD1
Expression
C
Macaque
Expression Level
GNB4+_5
GNB4+_7
GNB4+_1
GNB4+_0
GNB4+_2
GNB4+_4
GNB4+_16
GNB4+_8
GNB4+_11
GNB4+_3
GNB4+_13
Mouse
Expression Level
GNB4+_2
GNB4+_9
GNB4+_22

Figure S1: Correlation matrix for all genes. Pearson correlation table for expression of all 12 transcripts across clustered claustrum cells.

Figure S2: Opioid receptor expression in the male and female claustrum. Box plots comparing percent area coverage (PAC) of each opioid receptor gene within each cell cluster. Unpaired Wilcox test was used to compare expression levels between males and females. ns = not significant, *=p<0.05.

Figure S3: Opioid receptor expression in the male and female dorsal endopiriform. A) Expression levels of opioid receptors present in each endopiriform cell cluster within both male and female images. B) Box plots comparing percent area coverage (PAC) of each opioid receptor gene within each cell cluster. Unpaired Wilcox test was used to compare expression levels between males and females. ns = not significant.

Figure S4: Opioid receptor expression using sn-RNA seq data of mouse claustrum. (A) UMAP based representation of opioid receptor expression within all identified mouse claustrum neuron populations. Cells coloured based on level of expression represented through Pearson residuals. Excitatory clusters labelled in red, inhibitory clusters labelled in blue. (B) Gene expression heatmap of both opioid receptors and select population markers within identified neuronal populations down sampled to 1000 cells. (C) Violin plots representing the expression of *Oprk* within all identified GLUT_GNB4+ subclusters within the macaque (top) and mouse (bottom).
